# A Rare Case of Phentermine-Induced Nonischemic Cardiomyopathy

**DOI:** 10.7759/cureus.31114

**Published:** 2022-11-05

**Authors:** Devanshi Damani, Mariam Hassan, Swati Mahapatra, Swathi Prakash, Sara Alhariri, Jared Bies, Bhavi Trivedi, Brian P Edwards

**Affiliations:** 1 Internal Medicine, Texas Tech University Health Sciences Center El Paso, El Paso, USA; 2 Internal Medicine, Texas Tech University Health Sciences Center El Paso, El Paso , USA

**Keywords:** anorexigen, phentermine-topiramate, weight loss counselling, obesity and overweight, heart failure with reduced ejection fraction, drug-induced cardiomyopathy

## Abstract

Obesity is a global epidemic with steadily increasing prevalence in most countries. Weight loss is generally challenging for patients to tackle in the face of the temptation to overeat and avoid physical activity. Hence, clinicians and patients alike are likely to steer toward the use of *anorexigens*. We report the case of a 33-year-old female with no significant cardiac history who presented with dyspnea, productive cough, and chest pressure for one month and was diagnosed with new-onset heart failure with a reduced ejection fraction secondary to prolonged phentermine use. The authors aim to highlight phentermine’s potential for precipitating heart failure, even in a young, relatively healthy person, especially with a growing obese population. Ultimately, healthy weight loss can be achieved by implementing dietary changes and encouraging adequate physical activity, as the World Health Organization (WHO) recommended. Anorectic drugs may be employed for short-term use. Further research concerning the long-term side effects of phentermine may avert the prescriber and patient from abusing this drug.

## Introduction

Obesity is a well-established global epidemic with steadily increasing prevalence in most countries. The World Health Organization (WHO) defines obesity as a body mass index (BMI) of 30 kg/m2 or greater [1]. Obesity promotes the incidence of cardiovascular risk factors, including type 2 diabetes mellitus, hypertension, and hyperlipidemia. More than two in five adults (42.4%) are obese [2]. As time passes, weight loss is becoming more challenging for patients to tackle in the face of the temptation to overeat and the avoidance of physical activity. Hence, all health professionals must be familiar with the guidelines of various weight loss-directed interventions. This paper focuses on the utility and an underreported detrimental effect of phentermine.

Phentermine is structurally analogous to amphetamine [3]. It stimulates the hypothalamic release of norepinephrine and epinephrine, which contribute toward phentermine’s appetite suppressant property and abuse potential [3]. The U.S. Food and Drug Administration (FDA) first approved the use of phentermine in 1959 “as a short-term adjunct (a few weeks) in a regimen of weight reduction based on exercise, behavioral modification, and caloric restriction” for managing exogenous obesity [4]. Despite this instruction, it is frequently prescribed off-label for longer periods.

An insightful literary review reveals that the effects of long-term phentermine monotherapy on weight and cardiovascular disease are limited to a few case reports. The authors, however, postulate that chronic exogenous exposure to a sympathomimetic is cardiotoxic, and this is exemplified by Takotsubo cardiomyopathy, which is also provoked by high levels of catecholamines [5]. The case below unveils phentermine’s potential for precipitating devastating nonischemic cardiomyopathy in a 33-year-old obese female without significant underlying cardiovascular disease.

## Case presentation

A 33-year-old female with a past medical history of generalized anxiety disorder presented to the emergency room for worsening shortness of breath for one month. It was associated with a productive cough (clear-yellow sputum) and intermittent chest pressure that was non-radiating. A review of systems was otherwise negative. Her symptoms were exacerbated by laying down, for which she had begun sleeping in a recliner. The patient reported having had to sweep out storage space one month ago and believes this activity had precipitated her dyspnea. She also mentioned having discontinued phentermine-HCl 15mg once daily two months prior. The patient had been taking this drug for more than two years for weight loss. She denied taking other medications, including alcohol, tobacco, or illicit drugs.

On admission, the patient’s vitals were blood pressure 142/95, heart rate 120, respiratory rate 20, and 94% saturation on room air. The physical examination was remarkable for morbid obesity (calculated BMI 46.5kg/m^2^, Class 3), crackles in both lung bases, and trace pitting edema of both lower extremities. She did not have labored respiration, jugular venous distention, ascites, or S3 or S4 heart sounds. Laboratory work-up revealed the findings presented in Table 1.

**Table 1:** Laboratory workup results The laboratory results upon admission to the hospital. The reference range for each is presented in the third column. POCT: Point-of-Care Testing

Tests	Results	Normal range
White blood count (WBC)	6.53 × 10^3^/UL	4.50-11.00 × 10^3^/UL
Red blood count (RBC)	5.19 × 10^6^/UL	3.50-5.50 × 10^6^/UL
Hemoglobin	12.2 g/dL	12.0-15.0 g/dL
Platelets	349 × 10^3^/UL	150-450 × 10^3^/UL
Sodium, serum	137 mmol/L	135-145 mmol/L
Potassium, serum	3.7 mmol/L	3.5-5.1 mmol/L
Chloride, serum	109 mmol/L	98-111 mmol/L
Bicarbonate	20 mmol/L	20-29 mmol/L
Glucose	106 mg/dL	70-100 mg/dL
Blood urea nitrogen, serum	9 mg/dL	7-22 mg/dL
Magnesium, serum	1.7 mg/dL	1.6-2.3 mg/dL
Phosphorus, serum	2.2 mg/dL	2.5-4.5 mg/dL
Thyroid-stimulating hormone (TSH)	0.713 mIU/L	0.465-4.680 mIU/L
Glycated hemoglobin A1c (HbA1c)	5.7%	<5.7%
Respiratory panel	Not detected	Not detected
Troponin (POCT)	<0.02 ng/mL	0.00-0.08 ng/mL
B-type natriuretic peptide (BNP)	932 pg/mL	0-100 pg/mL
Alcohol level	<10 mg/dL	Negative
Urine drug screen (UDS)	Negative	Drug cutoff concentrations

An electrocardiogram (ECG) showed sinus tachycardia, and mild vascular congestion was appreciated on the chest X-ray. A chest computed tomography (CT) angiography was obtained for concern of a pulmonary embolus which was ruled out. The CT angiography, however, was suggestive of severe congestive heart failure (HF) and bibasilar edema. The patient was started on daily intravenous furosemide.

A transthoracic echocardiogram (TTE) revealed a mildly dilated left ventricle, with severely reduced left ventricular systolic function and global hypokinesis. The ejection fraction was estimated to be less than 20%. The right ventricle was grossly normal in size, with normal systolic function and without evidence of pulmonary hypertension. Mild-to-moderate mitral regurgitation could be appreciated. Subsequently, the patient underwent a left heart catheterization (LHC) which was notable for mild, non-obstructive epicardial coronary artery disease.

The patient’s dyspnea, cough, and tachycardia improved with continued diuresis. Given her new-onset heart failure with a reduced ejection fraction (HFrEF) diagnosis, she was promptly started on guideline-directed medical therapy and advised to follow up in the cardiology clinic as an outpatient. In an attempt to pursue the underlying etiology of this patient’s cardiomyopathy, a phentermine level was ordered, which was negative.

## Discussion

Our patient is a young, obese female who was evaluated for progressively worsening dyspnea, chest discomfort, and clear, productive sputum for one month. While asthma is high on the differential list, a lack of a prior history of asthma, allergies, or family history makes the diagnosis less likely. Documented tachycardia, borderline tachypnea, appreciable bilateral basal crackles, trace lower extremity pitting edema, and reported orthopnea was more concerning for HF. The finding of vascular congestion on the chest radiograph, pulmonary edema on the CT scan, elevated BNP, and the patient’s clinical improvement with furosemide treatment supported the clinical diagnosis of HF. An Echocardiographic (ECHO) examination revealed an ejection fraction of less than 20%, reduced left ventricular function, mild-to-moderate mitral regurgitation, and global hypokinesis, confirming HF. Other considerable causes of subacute dyspnea, such as anemia, sarcoidosis, interstitial lung disease, pneumonia, myocarditis, and pericarditis, were less likely given her normal hemoglobin level, lack of perihilar lymphadenopathy, negative CT scan for interstitial disease, the absence of any consolidation on imaging, a history negative for fever, chills, current or recent viral illness, as determined by the negative respiratory panel, including SARS-CoV-2 testing.

Heart failure with preserved ejection fraction (HFpEF) and HFrEF vary substantially in their etiology and pathophysiology [6-8]. Given our patient’s young age and obesity, HFpEF would likely be expected [7]. However, the new onset of HFrEF prompted further investigations. The patient denied being previously diagnosed with any cardiac conditions, and an ECHO was negative for significant valvular abnormalities, which ruled out valvular pathologies precipitating the HF. A normal TSH level made metabolic causes of impaired cardiac function less likely. A lack of significant alcohol consumption or the use of illicit drugs and a negative UDS reduced the probability of toxic cardiomyopathy. A left heart catheterization ruled out significant coronary disease and global hypokinesis, excluding ischemic cardiomyopathy. On further probing, she revealed that the patient had been on phentermine for at least two years to lose weight and discontinued the medication about a month before her presentation. This prolonged duration of use, extending beyond the FDA’s recommendation of “a few weeks,” could have led to cardiotoxicity, resulting in HFrEF [4].

Obesity is a growing public health concern associated with increased morbidity and mortality [3]. Hence, physicians routinely encourage a healthy BMI by combining dietary modifications, physical activity, and supplement pharmacotherapy for patients with more obstinate weight loss journeys. Some of these drugs’ efficacy and safety profile led to their withdrawal from the market, with one significant concern being their risk for cardiotoxicity [3].

Phentermine is the most commonly prescribed drug for short-term weight loss [3]. It is largely excreted in the urine (62-85% unchanged) with a half-life of 19-24 hours [9]. Thus, our patient’s negative UDS and blood phentermine level could be explained by its short half-life and a history of drug discontinuation about two months ago. Although the acute, sympathomimetic adverse effects of phentermine are well-known, there appears to be a paucity of publications that delve into the repercussions of long-term use [10-12].

Bang WD et al. showcased a link between phentermine and pulmonary hypertension in a young female evaluated for shortness of breath while on phentermine for 35 days [13]. Another case reported by Singal AK et al. described a young bodybuilder being worked up for an embolic stroke. Initial discontinuation of phentermine improved cardiac function, followed by worsening ventricular function on spontaneous re-initiation of the medication three months later [14]. Phentermine has been associated with valvular abnormalities, atrial fibrillation, and more so when combined with fenfluramine or dexfenfluramine, resulting in market withdrawal [11, 15]. Conversely, a recent study using electronic health record data showed that long-term phentermine use (>12 months) was associated with minimal risk (0.3%) of cardiovascular disease [16].

The mechanism of cardiotoxicity of phentermine remains unclear. Being a sympathomimetic agent, it can cause tachycardia and provoke tachycardia-induced cardiomyopathy [17]. The reversibility of ensuing impaired cardiac function is also uncertain. While Singal AK et al. described improvement with discontinuation of therapy, further large-scale studies are needed to investigate the long-term side effects of this anorectic and its impact on cardiac health [13].

## Conclusions

Phentermine should be used restrictedly as an anorexigenic in patients with a BMI of 30kg/m^2^ or more with finite data about long-term sequelae. Although clinicians prefer phentermine because it is quick and effective, it wields a risk for long-term abuse. Through this case report, the authors aim to draw attention to a potential understated, cardiotoxic effect of phentermine. Large-scale studies are paramount to investigating this anorectic and its impact on cardiac health. Adequate counseling concerning an appropriate duration of use and possible cardiotoxicity prior to commencement is advised.
